# Inverse autonomic stress reactivity in depressed patients with and without prior history of depression

**DOI:** 10.1192/j.eurpsy.2021.870

**Published:** 2021-08-13

**Authors:** A. Agorastos, A. Heinig, K. Wiedemann, C. Demiralay

**Affiliations:** 1 Ii. Dept. Of Psychiatry, Aristotle University of Thessaloniki, Thessaloniki, Greece; 2 Psychiatry & Psychotherapy, University Medical Centre Hamburg-Eppendorf, Hamburg, Germany

**Keywords:** recurrent episode, history of depression, Depression, heart rate variability

## Abstract

**Introduction:**

There is a considerable association between major depressive disorder (MDD) and cardiovascular disease, most possibly relying on abnormalities in the autonomic nervous system (ANS)-related cardiac reactivity, although the exact underlying pathophysiological pathway is unclear.

**Objectives:**

This study tends to shed some additional light on this background by investigating ANS reactivity in MDD with respect to previous depression history through an objective stress challenge paradigm.

**Methods:**

The study assessed the effects of an overnight hypothalamus-pituitary-adrenal (HPA) axis stimulation with metyrapone (MET) on baseline ANS activity through linear and non-linear heart rate variability (HRV) measures in the morning of two continuous days in a group of 14 physically healthy, antidepressant-free patients with clinical, non-psychotic MDD, to investigate differences in autonomic reactivity with respect to prior MDD history.

**Results:**

The main findings of this study include statistically significant time x group interactions with respect to several HRV measures, suggesting substantial differences on autonomic reactivity between patients with and without depression history. Hereby, recurrent-episode MDD patients showed lower vagal activity, while first-episode MDD patients increased PNS activity after HPA axis stimulation.

**Conclusions:**

These findings indicate that HPA axis stimulation in MDD patients leads to inverse vagal response according to MDD history. We suggest that chronic stress system overactivation, as found in MDD, might lead to a progressive inversion of the original stress response through HPA axis and ANS divergence over the course of a recurrent illness. HRV could, thus, represent a significant biomarker in MDD with temporal sensitivity.

